# Sexual dimorphism in synaptic inputs to the mouse amygdala and orbital cortex

**DOI:** 10.3389/fnins.2023.1258284

**Published:** 2023-10-12

**Authors:** Etay Aloni, Muhammad Tibi, Hannah Hochgerner, Amit Zeisel

**Affiliations:** Faculty of Biotechnology and Food Engineering, Technion - Israel Institute of Technology, Haifa, Israel

**Keywords:** brain, mice, sexual dimorphism, tracing, connectivity, amygdala, orbital cortex, bed nucleus of the stria terminalis

## Abstract

The medial amygdala (MeA) is a sexually dimorphic brain region that regulates fear responses, emotional memories, and social behaviors. It is known to be larger and contains more cells in males. The MeA integrates information through input connections from olfactory regions, bed nucleus of the stria terminalis, ventral hippocampus, and thalamic and hypothalamic structures. We hypothesize that in addition to the size differences, there are differences in regional connectivity between the sexes. In this study, we utilized G-deleted rabies monosynaptic retrograde tracing to compare amygdala presynaptic cells in male and female whole mouse brains. We report differences in connection patterns to the amygdala, with higher overall connectivity (presynaptic per starter) in males and a larger fraction of inputs originating from the bed nucleus of the stria terminalis, lateral septum, and medial preoptic area. Furthermore, we examined input connections to the orbital cortex (ORB), a brain region shown to be larger in volume in females, and found the opposite trend, where females had more total inputs. Together, our findings extend the evidence for sexual dimorphism in the brain to the neuronal wiring pattern, with likely impacts on behavior and disease susceptibility.

## Introduction

Sexual dimorphism, the differences in traits between males and females of the same species, has been a focus of evolutionary and ecological biology for many years. Although these differences may explain variations in behavior and disease susceptibility between sexes, many animal studies perform experiments on single sex with high preferences for males, or when experiments were done on both sexes, the data were not analyzed by sex (Karp et al., 2017). The medial amygdala (MeA) is a bilateral region in the mammalian brain that plays a key role in regulating fear responses and emotional memories, and it is involved in many innate social behaviors (Petrulis, 2020). Human studies, mainly based on magnetic resonance imaging, reported higher amygdala volume in male compared to female brains (Goldstein et al., 2001; Lotze et al., 2019; DeCasien et al., 2022). Rodent studies revealed sexual differences, especially in the MeA, including differences in size, cell number, cell size, cell type distribution, gene expression, biophysical signatures, and synaptic organization (Cooke and Woolley, 2005; Xu et al., 2011; Chen et al., 2019; Matos et al., 2020; Elkind et al., 2023). Subterritories of the MeA (anterior, posteroventral, and posterodorsal) receive input from various brain regions, including the olfactory bulb (OB), piriform cortex (PIR), bed nucleus of the stria terminalis (BST), CA1 in the ventral hippocampus, and thalamic and hypothalamic structures (Keshavarzi et al., 2014; Cádiz-Moretti et al., 2016; Fu et al., 2020). Although inputs of the MeA have been studied using retrograde tracing, connectivity differences between the sexes, particularly in the MeA, are not well-understood. In this study, we sought to expand our understanding of sex differences by examining the distal connections of neurons across the full volume of the brain (~+3.5, −4.5 mm AP from the bregma). We focused on two areas that are sexually dimorphic in cytoarchitecture, namely, the MeA and the ORB. For this, we compared male and female mice injected with the ΔG rabies virus, which is used for monosynaptic retrograde tracing.

## Materials and methods

### Animals and ethics

In this study, we used 26 young adults, male and female C57Bl/6 mice (Jackson Laboratories), aged 8–10 weeks at the time of AAV injections. Ages were not significantly different between sexes (MeA: males, P58.6; females, P59.4 and ORB: males, P67.5; females, P59), and the average weight was 20.4 ± 1 g for females and 23.8 ± 1 g for males. The mice were group housed in standard cages (3–4 mice per cage) in a specific pathogen-free (SPF) animal facility. They were maintained on a 12/12-h day/night cycle, with *ad libitum* access to food and water. All experimental procedures were approved and performed under the legislation of the National Institute of Health guidelines and the Institutional Animal Care and Use Committee (IACUC).

### Virus injections for retrograde monosynaptic tracing

All surgeries were performed in an SPF room under strict aseptic conditions. Prior to each surgery, all surgical tools were sterilized. The mice were weighed, anesthetized with isoflurane (3%), and head-fixed in a stereotaxic apparatus (Drill and Microinjection Robot, Neurostar, Malvern, PA, USA). The anesthesia was maintained at a level of 1.4–1.8% isoflurane using a specialized small animal anesthetic device (Somnosuite, Kent Scientific, Torrington, CT, USA). The level of anesthesia was continuously monitored using a hind paw-fixated pulse oximeter, which measured oxygen saturation and heart rate. To maintain the body temperature at 37°C, a body temperature probe connected to a thermoregulated heating pad was used. Additionally, during the surgery, a local injection of lidocaine (4 mg/kg) was given to the drilling area for pain management. Subcutaneous administration of buprenorphine (0.05–0.1 mg/kg) was given for immediate analgesic effects after the surgery. After hair removal and exposing the scalp, two small holes were drilled in the scalp, and AAV (ssAAV-2/2-hSyn1-hHBbI/E-TVA950_mCherry_2A_oG-hGHp(A) 2 × 10^12^ vg/ml, VVF Zurich, v634-2, 200 nl) was bilaterally injected into either the medial amygdala (MeA, Bregma AP: −1.58; ML: ±2.2; DV: 5.6) or the orbital cortex (ORB, Bregma AP: 2.6; ML: ±0.6; DV: 2.6). Mice recovered from anesthesia in a heated chamber and returned to group housing in home cages, with post-surgery monitoring and administration of the painkiller buprenorphine (0.05–0.1 mg/kg) as necessary. After 3 weeks of AAV injection, the same stereotactic surgery was repeated for injections of rabies virus (pSADB19dG-GFP, 4.2 × 10^8^ IU/ml, VCF Charite, 200 nl), to the same coordinates.

### Tissue harvest and processing

Seven days after the second surgery, at the end of the experimental timeline, the mice were perfused for tissue collection. The mice were euthanized via intraperitoneal injection with an overdose of ketamine and xylazine (100 and 10 mg/kg, respectively). The mice were transcardially perfused with phosphate-buffered saline (PBS), followed by freshly prepared paraformaldehyde (PFA) at 4%. The brains were removed and kept in a PFA 4% solution at 4°C for 24 h, then transferred to PBS containing 0.05% sodium azide at 4°C for further use. For cryoprotection, the brains were transferred to 30% sucrose in PBS for 48 h before being embedded in OCT (Scigen). Coronal sections of 50 μm thickness were then cut on a cryostat (NX50, Thermo Scientific), counterstained with Hoechst (Trihydrochloride, Rhenium), mounted on slides, and imaged at three excitation wavelengths (378, 474, and 554 nm) using a 4X LAMBDA, N.A. 0.20, WD 20 mm objective on a Ti2-E Nikon microscope.

### Mouse brain atlas registration and region-wise cell quantification

For consistent alignment and quantification of full-volume brains, we developed accessible MATLAB-based graphical user interface software that can perform a complete histology analysis and statistics pipeline of mouse brain sections. It can be used to preprocess images, interactively register and align coronal slices to the Allen Mouse Brain Atlas (Allen Reference Atlas, 2023), count marked cells using adjustable parameters such as cell size, roundness, and fluorescence intensity (signal to noise ratio), perform data quality checks, and do statistics (https://github.com/muhammadtibi/Sexual_Dimorphism_Mosue_Brain). Images of coronal sections with fluorescently labeled cells were obtained for each hemisphere, and each hemisphere was analyzed separately. We aligned each mask to its corresponding histology slice using a semi-automated process based on manual user input of selected matching points on both the reference slice and the histological slice. After alignment, we segmented starter and presynaptic cells by defining cell outlines based on fluorescence channels, i.e., mCherry (AAV) and GFP (RV). The overlap distance between GFP and mCherry centroids of detected cells determined whether the cell was considered a starter cell. GFP+ and mCherry+ cells were counted to quantify the number of presynaptic and starter cells present in each region of the mouse brain. We restricted this analysis to Bregma ~+3.5, −4.5 mm; which means the olfactory bulb and cerebellum were omitted from the analysis due to difficulties in obtaining consistent and high-quality data at the edges of the brain. Finally, we established a data quality control pipeline that allowed a review of the extracted, relevant ROI, exploration of counts of cells, and the co-expression between them. Within the analyzed set of 36 MeA and 16 ORB-injected hemispheres, data quality checks were applied, ensuring a minimum of 20 detected starter cells and that over 60% of these cells were located in the amygdala for MeA and in the ORB for ORB hemispheres. Following these checks, 14 MeA hemispheres (*n* = 7 males, and *n* = 7 females) and 8 ORB hemispheres (*n* = 4 males and *n* = 4 females) met the criteria. Subsequent analyses were conducted only on regions averaging more than 30 presynaptic cells in either male or female samples. These rigorous quality criteria, leading to considerable data exclusion, were necessary due to difficulties in precisely reproducing injection sites, and we acknowledge this as a limitation of the study. The starter distribution was calculated by dividing the number of starters in each region by the total number of starters in the sample. Presynaptic per starter (PPS) for each region was calculated by dividing the number of presynaptic cells in that region by the total number of starters in the sample. The fraction per region (FPR) for each region was calculated by dividing the number of presynaptic cells in that region by the total number of presynaptic cells in the sample.

### Statistical analysis

For group comparison, male vs. female, we used the non-parametric Wilcoxon–Mann–Whitney test (*p*-value in the text). When a single hypothesis was tested, the null hypotheses were rejected when the *p-*value was <0.05. To control for multiple tests of brain regions, we additionally used the false discovery rate (FDR) of 5% (*q*-value in the text). Additional statistical results are listed in Supplementary Table 1. An overview of all statistical tests is provided in Supplementary Table 2.

## Results

**Figure 1:**
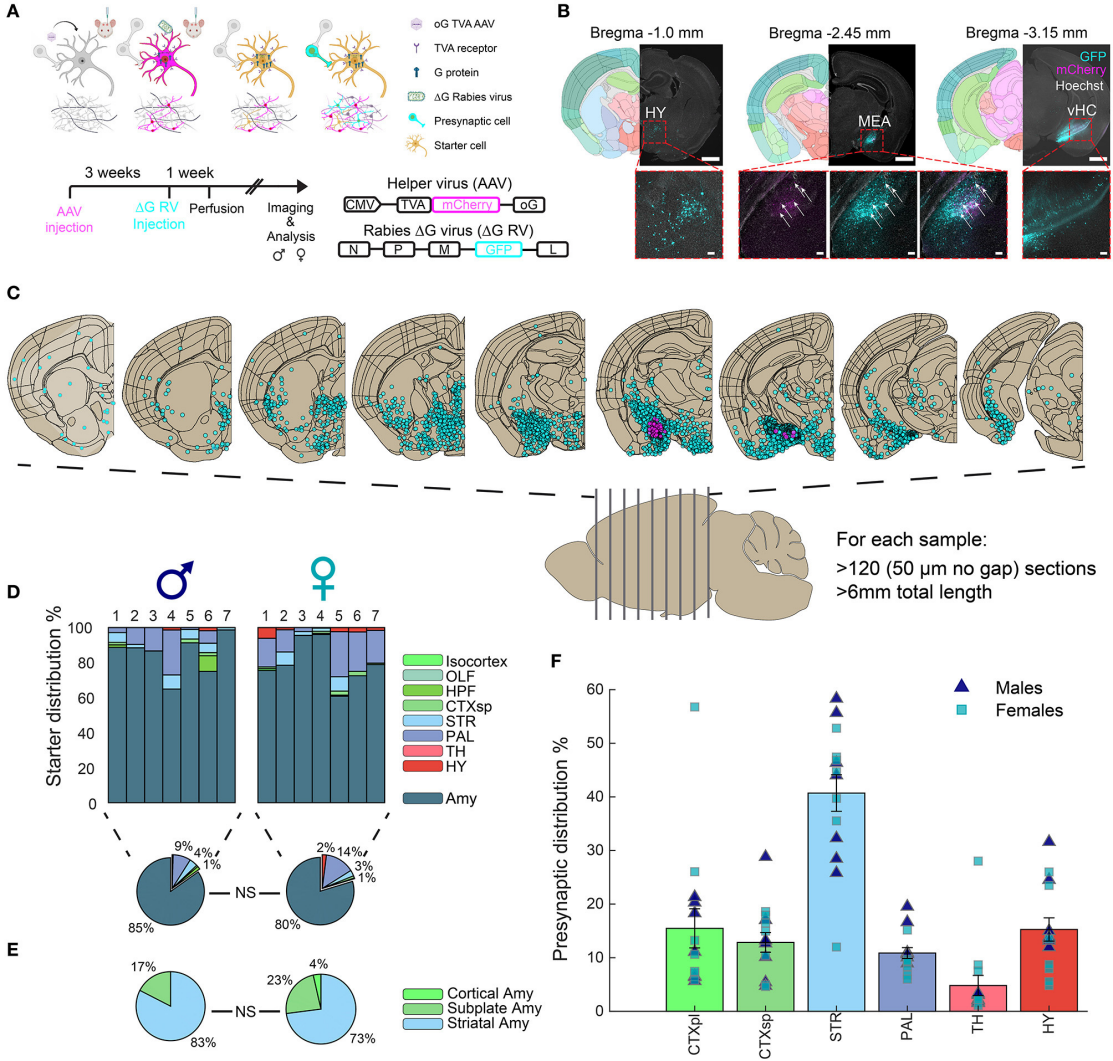
Monosynaptic inputs to the amygdala**. (A)** Experimental outline, mechanisms, and timeline for ΔG-rabies monosynaptic tracing: A helper virus with oG and TVA genes is injected into the mouse brain. After 3 weeks, infected cells express oG and TVA proteins. The EnvA-ΔG-rabies virus is then injected into the same site. This virus requires the TVA receptor to enter cells and the oG protein for retrograde transfer to presynaptic cells. Thus, ΔG-rabies virus can retrogradely cross only from AAV-injected cells. Image designed by BioRender.com. Virus details are in the bottom right. **(B)** Monosynaptic retrograde tracing images in three representative coronal hemispheres, alongside registered brain atlas reference sections (atlas.brain-map.org). AAV+ cells, magenta; presynaptic cells, cyan; double positive starter cells indicated with white arrows. Scale bars; overview, 1 mm; zoom-ins (below) to hypothalamus (HY), ventral hippocampus (HC) and MeA, 100 μm. **(C)** The distribution of registered cells in coronal sections along the a.-p. axis of the mouse brain. **(D)** Distribution of identified starter cells in males (*n* = 7) and females (*n* = 7); average per sex in pie charts (below). **(E)** Distribution of starter cells in the main amygdala territories, with no significant differences between sexes. **(F)** Distribution of identified presynaptic cells (mean ± SEM) in major brain regions for both male and female samples.

In this study, we aimed to investigate sex-specific differences in monosynaptic input connections to the MeA in young adult mice. We administered bilateral MeA injections to 8 male and 10 female mice with the AAV helper virus (expressing optimized glycoprotein (oG), avian receptor (TVA), and mCherry), followed by injections of the G-deleted (ΔG) SAD-B19 rabies virus (GFP) (Figure 1A). Coronal brain sections were imaged and analyzed, and hemispheres with verified expression in the injection site were included in subsequent analysis (Figures 1B, C). Although small-volume injections were aimed at the MeA, small amounts of starter cells (i.e., AAV-RV double-positive cells) were also detected in other amygdala subregions (Figures 1D, E), and henceforth, we refer to the amygdala as the injection site. Importantly, no significant difference was found in the injection sites between males and females. Following imaging, we registered the coronal sections to the Allen Mouse Brain Atlas (AMBA) and quantified starter cells and presynaptic cells for each brain region (see the “Materials and Methods” section). Seven hemispheres in males and females each passed image and injection quality thresholds (see the “Materials and Methods” section). We found that the amygdala in both male and female mice was highly connected to most territories of the brain, with striatal (STR) regions containing more than 40% of MeA input cells and the cortical plates (CTXpl) and hypothalamus (HY) with ~15% of MeA input cells (Figure 1F).

### Sexual dimorphism in input fibers innervating the amygdala

**Figure 2:**
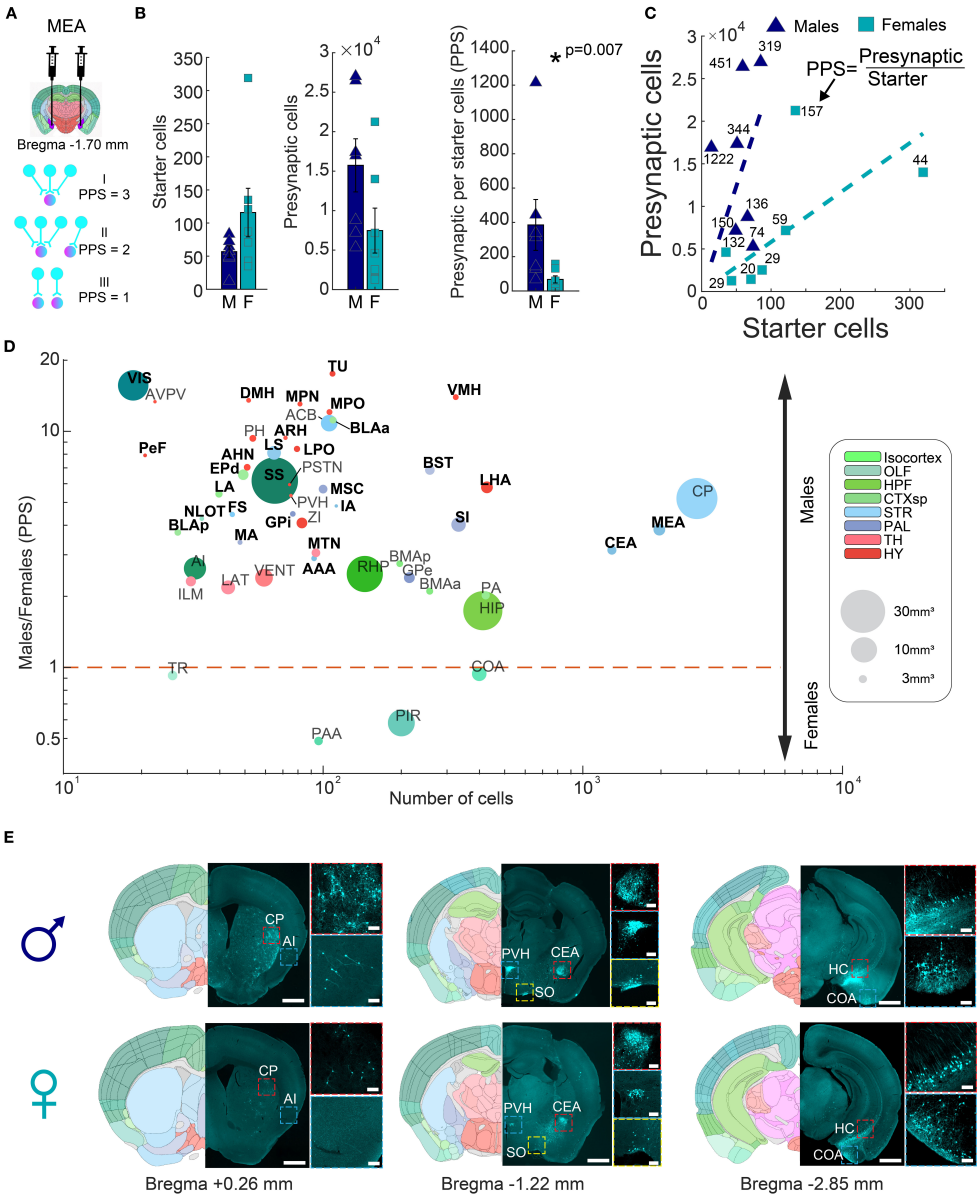
Connectivity sexual dimorphism in the amygdala of the mouse brain. **(A)** Illustration of injection sites in the MeA of helper and ΔG-rabies viruses on a mouse coronal section (upper panel), and three examples of presynaptic per starter (PPS) calculation (lower panel). Presynaptic cells are shown in magenta and starter cells in both magenta and pink colors. **(B)** Comparison of starter cell numbers, presynaptic cell numbers, and PPS between male and female samples (mean ± SEM). Asterisk indicates a significant difference (rank-sum test, *p* = 0.007, *n* = 7 males; *n* = 7 females). **(C)** Scatterplot showing the average PPS slope of male and female samples. **(D)** Scatterplot of brain regions based on the mean number of presynaptic cells in all samples on a logarithmic scale, as a function of the difference found in PPS in each of the regions between male and female samples on a logarithmic scale. Significantly different regions are written in bold black font (statistics in Supplementary Table 1). Each dot size corresponds to its region size. Colors match the Allen Mouse Brain Atlas regions color code. **(E)** Representative images showing differences in presynaptic cells in males (upper panels) and females (lower panels) for seven brain regions, in three coronal sections. Registered brain atlas reference sections (atlas.brain-map.org) are provided, and approximate bregma is indicated below. Scale bars; overview (left), 1 mm and zoom-in (right), 100 μm. Abbreviations for brain regions are listed in Supplementary Table 1.

To account for the variability of injection efficiencies between individual experiments, we next normalized the portion of presynaptic cells per starter (PPS). The PPS of a given region thus represents the connectivity “strength” of that region to the injected area (Figure 2A). We investigated sexual dimorphism in the input fibers innervating the mouse amygdala by comparing normalized counts of presynaptic cells (PPS) between males and females. Male mice had significantly higher input connections to the amygdala compared to females (385 ± 148.4 vs. 67.4 ± 20.7 PPS, respectively, rank-sum test, *p* = 0.007, *n* = 7 males; *n* = 7 females) (Figures 2B, C), indicating that on average, individual cells in the amygdala of males received input from a larger number of neurons. To test to what extent this phenomenon was common, or unique, to specific input areas, we performed per-region hypothesis testing of PPS between males and females. The set of analyzed regions included those that, on average, had at least 30 presynaptic cells in either males or females. Surprisingly, out of the 50 brain regions tested, 46 exhibited higher PPS in males (Fisher's exact test, *p* = 9.73 × 10^−8^). After considering multiple tests (FDR 5%), we identified 29 regions with significantly more presynaptic cells in males and none in females (Figure 2D, Supplementary Figure 1, rank-sum test, Supplementary Table 1). For example, males had more input from two cortical regions, namely, the somatosensory cortex (SS) and the visual cortex (VIS), and four cortical subplate regions, namely, the lateral amygdalar nucleus (LA), the basolateral amygdalar nucleus anterior and postireior parts (BLAa, BLAp) and the Endopiriform nucleus, dorsal part (EPd). The striatum (STR) had the highest number of presynaptic cells and six dimorphic input regions, including the central amygdala (CEA) and the MeA itself (Figures 2D, E), which indicated higher interconnectivity of the MeA in males compared to females. In the pallidum, five subregions had significantly higher PPS in males, including the BST and the substantia innominata (SI). The hypothalamus (HY) contained 10 small volume, dimorphic subregions, such as the ventromedial hypothalamic nucleus (VMH) and the arcuate hypothalamic nucleus (ARH), with higher PPS in males, as did the midline group of the dorsal thalamus (MTN) within the thalamus (TH). Finally, inputs from the olfactory system's nucleus of the lateral olfactory tract (NLOT) were significantly biased toward males. Importantly, we found no effect of laterality on amygdala connectivity; inputs to left and right hemispheres were similar (267 ± 161 vs. 183 ± 60 PPS, respectively, rank-sum test, *p* = 0.804, Supplementary Figure 2). In conclusion, our data revealed significant differences in the number of input connections to the amygdala between male and female mice, highlighting the importance of considering sex as a biological variable in the study of the amygdala and its associated behaviors.

### Sexual dimorphism in the input fibers innervating the ORB

**Figure 3:**
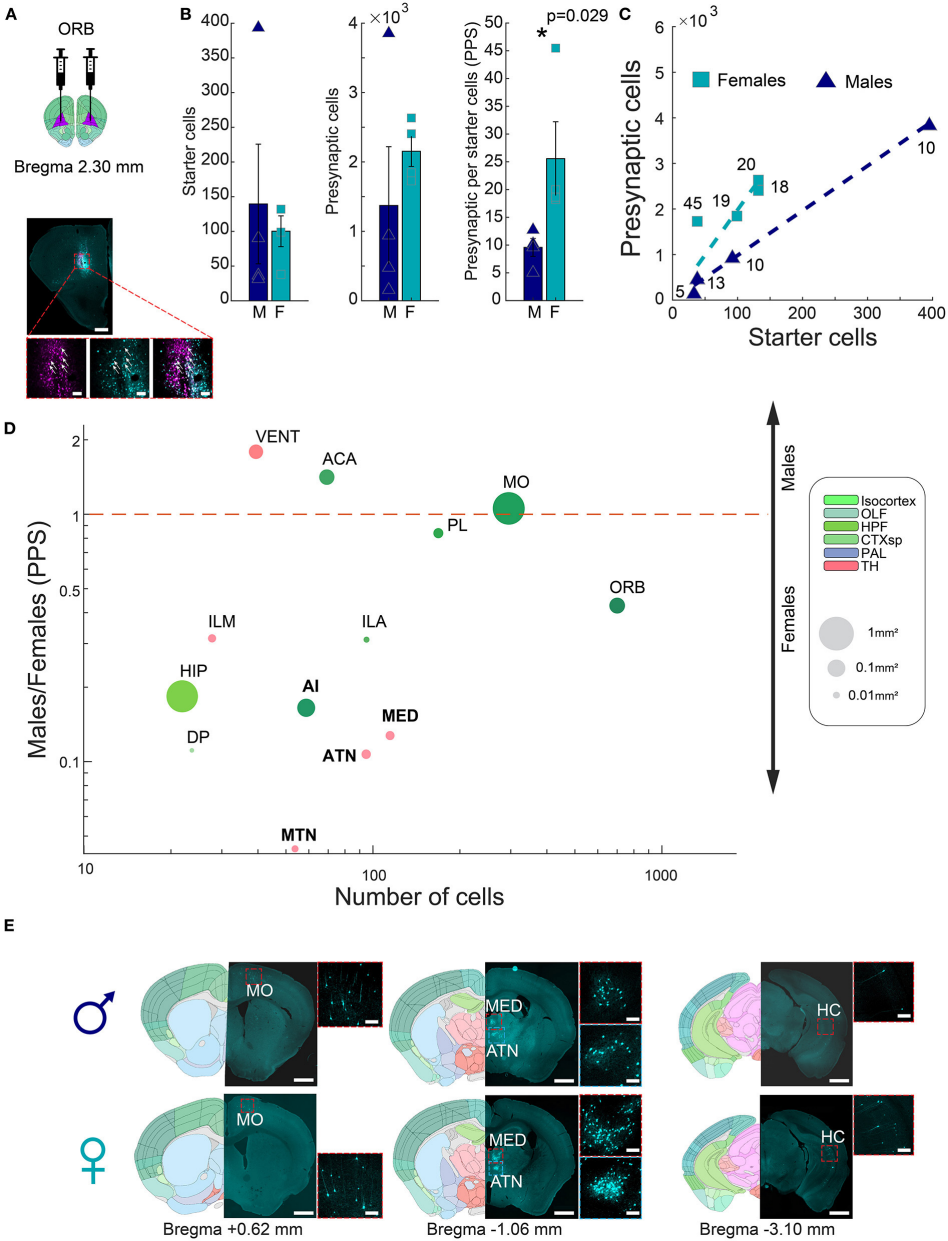
Connectivity sexual dimorphism in the ORB of the mouse brain. **(A)** Illustration of injection sites in the ORB of helper and ΔG-rabies viruses on a mouse coronal section (upper panel), and representative coronal section depicting monosynaptic retrograde tracing in the ORB (lower panel). AAV+ cells are shown in pink, presynaptic cells in magenta, and starter cells expressing both colors are marked with white arrows. Scale bars; overview (up), 1 mm, and zoom-in (below) 100 μm. **(B)** Comparison of starter cell numbers, presynaptic cell numbers, and PPS between males and females (mean ± SEM). Asterisk indicates a significant difference (rank-sum test, *p* = 0.029 *n* = 4 males; *n* = 4 females). **(C)** Scatterplot showing the average PPS slope of male and female samples. **(D)** Scatterplot of brain regions based on the mean number of presynaptic cells in all samples on a logarithmic scale, as a function of the difference found in PPS in each of the regions between male and female samples on a logarithmic scale. Significantly different regions are written in bold black font (statistics in Supplementary Table 1). Each dot size corresponds to its region size. The colors of regions match the Allen Mouse Brain Atlas regions color code. **(E)** Representative images showing differences in presynaptic cells in males (upper panels) and females (lower panels) for four brain regions, in three coronal sections. Registered brain atlas reference sections (atlas.brain-map.org) are provided, and approximate bregma is indicated below. Scale bars: overview (left), 1 mm and zoom-in (right), 100 μm. Abbreviations for brain regions are listed in Supplementary Table 1.

Our results above showed that the amygdala of males receives more input per neuron, correlating with previous evidence of a larger and denser medial amygdala in males. We next set out to test if a similar logic may hold in the opposite direction, in a region where female volume is higher. We and others (Anderson et al., 2013; Elkind et al., 2023) previously found that females have an increased volume of regions in the prefrontal cortex, and in particular, the ORB (Supplementary Figure 3). As before, we therefore performed G-deleted rabies monosynaptic retrograde tracing and input quantification to ORB and compared ORB presynaptic cells in male and female whole mouse brains (~+3.5, −4.5 mm AP from bregma, Figure 3A). Monosynaptic retrograde tracing in ORB detected overall smaller input networks (17.57 ± 4.3 PPS; *n* = 8), compared to the amygdala (225 ± 78.5 PPS, *n* = 14, rank-sum test, *p* = 0.0004). Between sexes, females had significantly more input connections to the ORB than males (25.57 ± 6.64 vs. 9.58 ± 1.6 PPS, respectively, rank-sum test, *p* = 0.029, *n* = 4 males; *n* = 4 females, Figures 3B, C). We normalized each hemisphere by dividing the presynaptic cells in each brain region by the number of ORB starter cells. As before, we first narrowed the set of analyzed regions to those with, on average, at least 30 presynaptic cells in either males or females. We found that out of 13 brain regions tested, 10 regions had more presynaptic cells in females, and only three regions had more presynaptic cells in males (Figure 3D, Supplementary Figure 4). One cortical region, the agranular insular area (AI), and three dorsal thalamic regions, namely, anterior group of the dorsal thalamus (ATN), midline group of the dorsal thalamus (MTN), and medial group of the dorsal thalamus (MED), had significantly different numbers of presynaptic cells between the sexes. Our findings suggest that sexually dimorphic areas in the brain could display a sexually dimorphic connectivity pattern as well. This underscores the importance of investigating sex differences in neuronal connectivity patterns in different brain regions.

### Distribution of presynaptic cells to the amygdala in the brain

**Figure 4:**
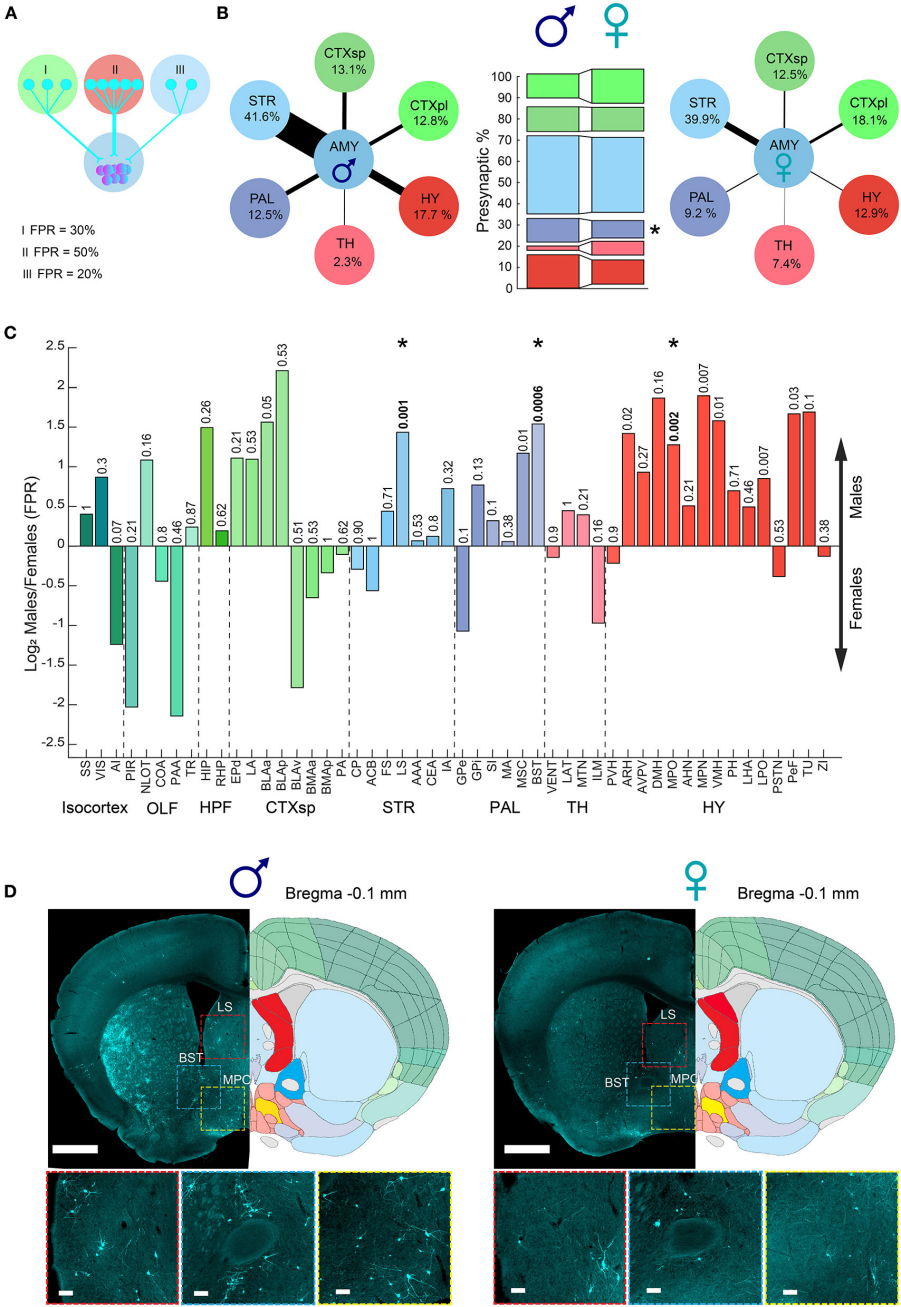
Sexual dimorphism in the amygdala connections fraction per region. **(A)** Illustration of three examples of fraction per region (FPR) calculation. **(B)** Diagram presenting a combination of PPS and FPR of meta brain areas (PPS and FPR represented by arrow size and percent, respectively) in males (left) and females (right). Comparison between meta brain areas FPR between males and females (middle bars), where the pallidum showed a significant sex difference. **(C)** Bar plot of brain regions showing difference in FPR between male and female samples presented as log2 of the proportion between the median of male and female. Colors match the Allen Mouse Brain Atlas regions color code. The abbreviations for brain regions are listed in Supplementary Table 1. **(D)** Example of three brain regions on top of two representative coronal sections alongside registered brain atlas reference sections (atlas.brain-map.org) showing differences in the number of presynaptic cells in males (left) and females (right). Scale bars, overview (up), 1 mm; zoom-ins (below), 100 μm. Abbreviations for brain regions are listed in Supplementary Table 1.

In addition to the overall trend of more cells innervating the amygdala in males (PPS), we next investigated whether there are sex differences in the individual contributions of different brain regions to signals arriving at the amygdala. We examined the presynaptic cells of each region as a percentage of the total presynaptic cells in the hemisphere, fraction-per-region (FPR, Figure 4A). For example, in “Male 1,” out of a total of 8,952 presynaptic cells, 304 presynaptic cells were in the hippocampus; therefore, the FPR of the hippocampus in that sample is 0.03 (304/8,952). In this way, the FPR analysis normalized the overall trend of more cells (PPS) innervating the amygdala in males. Our results revealed sex differences in the distribution of presynaptic cells innervating the amygdala area (Figure 4). Specifically, the amygdala in males received a greater fraction of inputs from the cortical subplate (CTXsp), PAL, and HY, while females received a higher fraction of inputs from the cortical plate (CTXpl) and thalamus (TH). The pallidum displayed a significant difference in the contribution of presynaptic cells innervating the amygdala between males and females (Figure 4B). Examining all brain regions after considering multiple tests, we found that the BST, LS, and MPO had a significantly higher contribution of presynaptic cells innervating the amygdala in males compared to females (Figures 4C, D). Interestingly, those three regions are known to be sexually dimorphic and all play key roles in social behaviors (Simerly and Swanson, 1986; Yahr et al., 1994; Lukas et al., 2013; Menon et al., 2021). In the ORB, the ATN and MTN regions had a significantly higher contribution of presynaptic cells. Overall, our results demonstrate sex bias in the connectivity to the amygdala, especially the MeA. Males had more PPS, and a larger fraction of inputs originated from the BST, LS, and MPO, while females had a larger fraction of inputs from the TH and the OLF. Overall, most regions that we found to have sex differences in their connectivity to the amygdala, such as the BST and MPO, were described to differ between the sexes in other traits, such as region size, cell density, or gene expression (Swaab, 1984; Wei et al., 2018; Elkind et al., 2023). Others, such as the thalamus and LS, displayed no sex differences in our previous analysis of region volumes and cell densities [Supplementary Figure 3, (Elkind et al., 2023)] or other studies, suggesting that their sex differences could be restricted to dimorphic connectivity patterns.

## Discussion

The aim of the present study was to investigate sex-specific differences in monosynaptic retrograde connections to the MeA. The results of this study align with previous research that has shown sex differences in the structure and function of the amygdala and the orbital cortex. For example, previous studies have shown sex-specific biophysical signatures with altering spiking-frequency adaptation (Matos et al., 2020), and others describe that the amygdala is larger in size and has more cells in males (Pfau et al., 2016), while the ORB is larger in size and has more cells in females (Anderson et al., 2013; Elkind et al., 2023). The current study expands on this research by demonstrating sex differences in the connectivity patterns of these brain regions. The results of the study demonstrated significant sex differences in the number and patterns of presynaptic cells innervating both the amygdala and ORB (Figures 2, 3). Specifically, we observed that males have a higher density of connections per cell in the amygdala compared to females. This finding is consistent with previous work in rats (Cooke and Woolley, 2005), which reported that a subregion of the MeA (MeApd) had higher levels of excitatory synapses per neuron and a higher miniature EPSC (mEPSC) frequency in males compared to females. The rat study also reported lateralization differences between left and right MeA, while no laterality difference was reported in mice (Pfau et al., 2016). Our results were in line with the later finding in mice, and we observed no difference in left and right connectivity using monosynaptic retrograde tracing (Supplementary Figure 2), providing additional evidence that not all mammals show lateralization in the amygdala. In addition to the differences in the amygdala, we found opposing biases in the ORB, with females receiving more inputs from distinct subcortical areas than males. This finding suggests that the sexually dimorphic areas in the brain may have sexually dimorphic connectivity patterns as well.

The BST and MeA are both part of the loosely defined limbic system, which is involved in emotional processing. The BST is involved in the regulation of anxiety-related behaviors (Lebow and Chen, 2016), while the MeA is involved in regulating fear responses and emotional memories (Petrulis, 2020). Dysregulation of the BST and MeA has been implicated in the pathophysiology of several neuropsychiatric disorders, including anxiety disorders, depression, and post-traumatic stress disorder (Davis et al., 2010; Lebow and Chen, 2016). Our finding that the BST had a significantly higher distribution of presynaptic cells in males compared to females suggests a potential role in the modulation or regulation of hormonal release and emotional and social masculine behaviors and may provide insight into the underlying neural mechanisms of the susceptibility of sexual dimorphism to neuropsychiatric disorders.

Overall, our study provides further evidence of sex differences in the connectivity patterns of the brain. The higher density of connections in the amygdala of males suggests that this region may play a more prominent role in male-specific behaviors, such as aggression and mating. The opposing bias in the ORB suggests that this region may play a more prominent role in female-specific behaviors, such as social interaction and emotion regulation. Future studies are encouraged to delve deeper into these findings to elucidate their potential impact on dimorphic sexual behaviors. Together, our findings suggest that sexually dimorphic brain regions may have different patterns of connectivity that underlie sex-specific differences in behavior and cognitive function. However, further studies are needed to fully understand the functional implications of these sex-specific connectivity patterns. Critically, the results of the study contribute to the growing body of research demonstrating the importance of considering sex as a biological variable in the study of the brain and its associated behaviors.

## Data availability statement

The raw data supporting the conclusions of this article will be made available by the authors, without undue reservation.

## Ethics statement

The animal study was approved by Technion Institutional Animal Care and Use Committee (IACUC). Technion, Israel. The study was conducted in accordance with the local legislation and institutional requirements.

## Author contributions

EA: Conceptualization, Data curation, Formal analysis, Investigation, Visualization, Methodology, Writing original draft, Writing review and editing. MT: Data curation, Methodology, Formal analysis, Software. HH: Conceptualization, Funding acquisition, Writing review and editing. AZ: Conceptualization, Funding acquisition, Supervision.

## References

[B1] Allen Reference Atlas (2023). Mouse Brain. Available online at: atlas.brain-map.org (accessed September 5, 2023).

[B2] Anderson L. C. Bolling D. Z. Schelinski S. Coffman M. C. Pelphrey K. A. Kaiser M. D.. (2013). Sex differences in the development of brain mechanisms for processing biological motion. Neuroimage 83, 751–60. 10.1016/j.neuroimage.2013.07.040 23876243 PMC3815992

[B3] Cádiz-Moretti B. Otero-García M. Martínez-García F. Lanuza E. (2016). Afferent projections to the different medial amygdala subdivisions: a retrograde tracing study in the mouse. Brain Struct. Funct. 221, 1033–65. 10.1007/s00429-014-0954-y 25503449

[B4] Chen P. B. Hu R. K. Wu Y. E. Pan L. Huang S. Micevych P. E.. (2019). Sexually dimorphic control of parenting behavior by the medial amygdala. Cell 176, 1206–1221. 10.1016/j.cell.2019.01.024 30773317 PMC6555485

[B5] Cooke B. M. Woolley C. S. (2005). Sexually dimorphic synaptic organization of the medial amygdala. J. Neurosci. 25, 10759–67. 10.1523/JNEUROSCI.2919-05.2005 16291949 PMC6725860

[B6] Davis M. Walker D. L. Miles L. Grillon C. (2010). Phasic vs. sustained fear in rats and humans: role of the extended amygdala in fear vs. anxiety. Neuropsychopharmacology 35, 105–35. 10.1038/npp.2009.109 19693004 PMC2795099

[B7] DeCasien A. R. Guma E. Liu S. Raznahan A. (2022). Sex differences in the human brain: a roadmap for more careful analysis and interpretation of a biological reality. Biol. Sex Differ. 13, 1–21. 10.1186/s13293-022-00448-w 35883159 PMC9327177

[B8] Elkind D. Hochgerner H. Aloni E. Shental N. Zeisel A. (2023). Sex-, strain and lateral differences in brain cytoarchitecture across a large mouse population. Elife. Available online at: https://elifesciences.org/articles/82376 (accessed September 5, 2023). 10.7554/eLife.82376 PMC10212558 37144870

[B9] Fu J. Y. Yu X. D. Zhu Y. Xie S. Z. Tang M. Y. Yu B.. (2020). Whole-brain map of long-range monosynaptic inputs to different cell types in the amygdala of the mouse. Neurosci. Bull. 36, 1381–94. 10.1007/s12264-020-00545-z 32691225 PMC7674542

[B10] Goldstein J. M. Seidman L. J. Horton N. J. Makris N. Kennedy D. N. Caviness V. S.. (2001). Normal sexual dimorphism of the adult human brain assessed by *in vivo* magnetic resonance imaging. Cereb. Cortex 11, 490–7. 10.1093/cercor/11.6.490 11375910

[B11] Karp N. A. Mason J. Beaudet A. L. Benjamini Y. Bower L. Braun R. E.. (2017). Prevalence of sexual dimorphism in mammalian phenotypic traits. Nat. Commun. 8, 15475. 10.1038/ncomms15475 28650954 PMC5490203

[B12] Keshavarzi S. Sullivan R. K. P. Ianno D. J. Sah P. (2014). Functional properties and projections of neurons in the medial amygdala. J. Neurosci. 34, 8699–715. 10.1523/JNEUROSCI.1176-14.2014 24966371 PMC6608208

[B13] Lebow M. A. Chen A. (2016). Overshadowed by the amygdala: the bed nucleus of the stria terminalis emerges as key to psychiatric disorders. Mol. Psychiatry 21, 450–63. 10.1038/mp.2016.1 26878891 PMC4804181

[B14] Lotze M. Domin M. Gerlach F. H. Gaser C. Lueders E. Schmidt C. O.. (2019). Novel findings from 2,838 adult brains on sex differences in gray matter brain volume. Sci. Rep. 9, 1–7. 10.1038/s41598-018-38239-2 30737437 PMC6368548

[B15] Lukas M. Toth I. Veenema A. H. Neumann I. D. (2013). Oxytocin mediates rodent social memory within the lateral septum and the medial amygdala depending on the relevance of the social stimulus : male juvenile vs. female adult conspecifics. Psychoneuroendocrinology 38, 916–926. 10.1016/j.psyneuen.2012.09.018 23102690

[B16] Matos H. Y. Hernandez-Pineda D. Charpentier C. M. Rusk A. Corbin J. G. Jones K. S.. (2020). Sex differences in biophysical signatures across molecularly definedmedial amygdala neuronal subpopulations. eNeuro 7, 1–15. 10.1523/ENEURO.0035-20.2020 32493755 PMC7333980

[B17] Menon R. Süß T. de Oliveira V. E. M. Neumann I. D. Bludau A. (2021). Neurobiology of the lateral septum: regulation of social behavior. Trends Neurosci. 45, 27–40. 10.1016/j.tins.2021.10.010 34810019

[B18] Petrulis A. (2020). Structure and function of the medial amygdala. 1st ed. Handb. Behav. Neurosci. 5, 7. 10.1016/B978-0-12-815134-1.00002-7

[B19] Pfau D. R. Hobbs N. J. Breedlove S. M. Jordan C. L. (2016). Sex and laterality differences in medial amygdala neurons and astrocytes of adult mice. J. Comp. Neurol. 524, 2492–502. 10.1002/cne.23964 26780286 PMC4900922

[B20] Simerly R. B. Swanson L. W. (1986). The organization of neural inputs to the medial preoptic nucleus of the rat. J. Comp. Neurol. 246, 312–42. 10.1002/cne.902460304 3517086

[B21] Swaab (1984). Sexually dimorphic. Science 228, 1112–5. 10.1126/science.3992248 3992248

[B22] Wei Y. C. Wang S. R. Jiao Z. L. Zhang W. Lin J. K. Li X. Y.. (2018). Medial preoptic area in mice is capable of mediating sexually dimorphic behaviors regardless of gender. Nat. Commun. 9, 648. 10.1038/s41467-017-02648-0 29348568 PMC5773506

[B23] Xu X. Coats J. K. Yang C. F. Wang A. Ahmed O. M. Alvarado M.. (2011). Modular genetic control of sexually dimorphic behaviors. Cell 148, 596–607. 10.1016/j.cell.2011.12.018 22304924 PMC3326403

[B24] Yahr P. Finn P. D. Hoffman N. W. Sayag N. (1994). Sexually dimorphic cell groups in the medial preoptic area that are essential for male sex behavior and the neural pathways needed for their effects. Psychoneuroendocrinology 19, 463–70. 10.1016/0306-4530(94)90033-7 7938347

