# Supplementary material for: Sexual dimorphism in synaptic inputs to the mouse amygdala and orbital cortex
Source: Front Neurosci. 2023 Oct 12;17:1258284. doi: 10.3389/fnins.2023.1258284 (PMC10601666; doi:10.3389/fnins.2023.1258284)
Supplement: Supplementary file 1 [file Data_Sheet_1.docx]

**Aloni et al.**

**Supplementary files legends**

**Supplementary Figure 1: Monosynaptic retrograde connections to the MeA. (**A) Number of presynaptic cells in the different brain regions in males and females (mean ± SEM). (B) Number of PPS cells in the different brain regions in males and females (mean ± SEM). Colors match the Allen Mouse Brain Atlas regions color code. n=7 males; n=7 females. Brain region abbreviations are listed in Supp. Table 1.

**Supplementary Figure 2: Connectivity lateralization in the mouse amygdala. (**A) Comparison of PPS (mean ± SEM) between right and left hemispheres. NS, non-significant. (B) Scatter plot comparing the average PPS slope between right and left hemispheres. (C) Scatter plot of brain regions based on the mean number of presynaptic cells in all samples on a logarithmic scale, as a function of the difference found in PPS in each region between left and right samples on a logarithmic scale. Each dot size corresponds to its region size, no region significantly differed between right and left hemispheres. Colors match the Allen Mouse Brain Atlas regions color code. n=7 right; n=7 left. pooled from males and females’ hemispheres (no significant left-right differences were found between the sexes). Brain region abbreviations are listed in Supp. Table 1.

**Supplementary Figure 3:** **Sex differences in regional neuroanatomy over a large mouse cohort.** Comparison of region volume, number of cells and cells density between males (n=271) and females (n=266), in five different regions of the mouse brain, using Elkind et al. [9] brain explorer <https://github.com/delkind/mouse-brain-cell-counting>). Asterisks indicate significant difference (t-test). Brain regions abbreviations are listed in Supp. Table 1.

**Supplementary Figure 4:** **Monosynaptic retrograde connections to the ORB.** (A) Number of presynaptic cells in the different brain regions in males and females (mean ± SEM). (B) Number of PPS cells in the different brain regions in males and females (mean ± SEM). Colors match the Allen Mouse Brain Atlas regions color code. n=4 males; n=4 females. Brain region abbreviations are listed in Supp. Table 1.

**Supplementary Table 1:** List of analyzed brain regions with abbreviations, and per-region quantification and statistics of presynaptic-per-starter (PPS) and fraction-per-region (FPR). Per comparison, dark grey indicates higher numbers in males, and light grey indicates higher number in females.

**Supplementary Table 2:** Summary of all statistical tests performed, per figure.
