# Supplementary material for: Sexual dimorphism in synaptic inputs to the mouse amygdala and orbital cortex
Source: Front Neurosci. 2023 Oct 12;17:1258284. doi: 10.3389/fnins.2023.1258284 (PMC10601666; doi:10.3389/fnins.2023.1258284)
Supplement: Supplementary file 3 [file Table_2.docx]

**Table S2**. Statistic tests and details by Figures:

| **Figure** | **Test** | **Sample size (n=)** | | **p / q** | **Comments** |
| --- | --- | --- | --- | --- | --- |
|  |  | **males** | **females** |  |  |
| 1D | Rank sum | 7 | 7 | By region, Table S1. All NS. | MEA. Sex comparison of the distribution of starter cells located in the amygdala. |
| 1E | Rank sum | 7 | 7 | By region, Table S1. All NS. | MEA. Sex comparison of the distribution of starter cells located in the amygdala. |
| 2B left | Rank sum | 7 | 7 | p=0.21 | MEA. Starter cells, whole brain. by sex comparison. |
| 2B middle | Rank sum | 7 | 7 | p=0.053 | MEA. Presynaptic cells, whole brain. by sex comparison. |
| 2B right | Rank sum | 7 | 7 | p=0.007, * | MEA. Presynaptic per starter cells (PPS), whole brain. by sex comparison. |
| 2D | Fisher’s exact test | 7 | 7 | p=9.73×10^-8^, * | MEA. Testing for nonrandom deviation of regions connectivity level (PPS) between sexes. |
| 2D | Rank sum, FDR test | 7 | 7 | By region, Table S1 * | MEA. MEA connectivity, all regions. By sex comparison. Accounting multiple tests, p and q values presented. |
| 3B left | Rank sum | 4 | 4 | p=0.67 | ORB. Starter cells, whole brain. by sex comparison. |
| 3B middle | Rank sum | 4 | 4 | p=0.34 | ORB. Presynaptic cells, whole brain. by sex comparison. |
| 3B right | Rank sum | 4 | 4 | p=0.029, * | ORB. Presynaptic per starter cells (PPS), whole brain. by sex comparison. |
| 3D | Fisher’s exact test | 4 | 4 | p=0.17 | ORB. Testing for nonrandom deviation of regions connectivity level (PPS) between sexes. |
| 3D | Rank sum | 4 | 4 | By region, Table S1, * | ORB connectivity, all regions. By sex comparison. |
| 4B | Rank sum | 7 | 7 | By region,  Table S1, NS | MEA. Distribution of presynaptic cells (FPR), meta brain areas. by sex comparison. |
| 4B | Rank sum | 7 | 7 | By region,  Table S1 * | MEA. Presynaptic per starter cells (PPS), meta brain areas. by sex comparison. |
| 4C | Rank sum FDR test | 7 | 7 | By region,  Table S1 * | MEA. Distribution of presynaptic cells (FPR), all regions. by sex comparison. Accounting multiple tests, p and q values presented. |
