# Supplementary figures and images for: Sexual dimorphism in synaptic inputs to the mouse amygdala and orbital cortex
Source: Front Neurosci. 2023 Oct 12;17:1258284. doi: 10.3389/fnins.2023.1258284 (PMC10601666; doi:10.3389/fnins.2023.1258284)

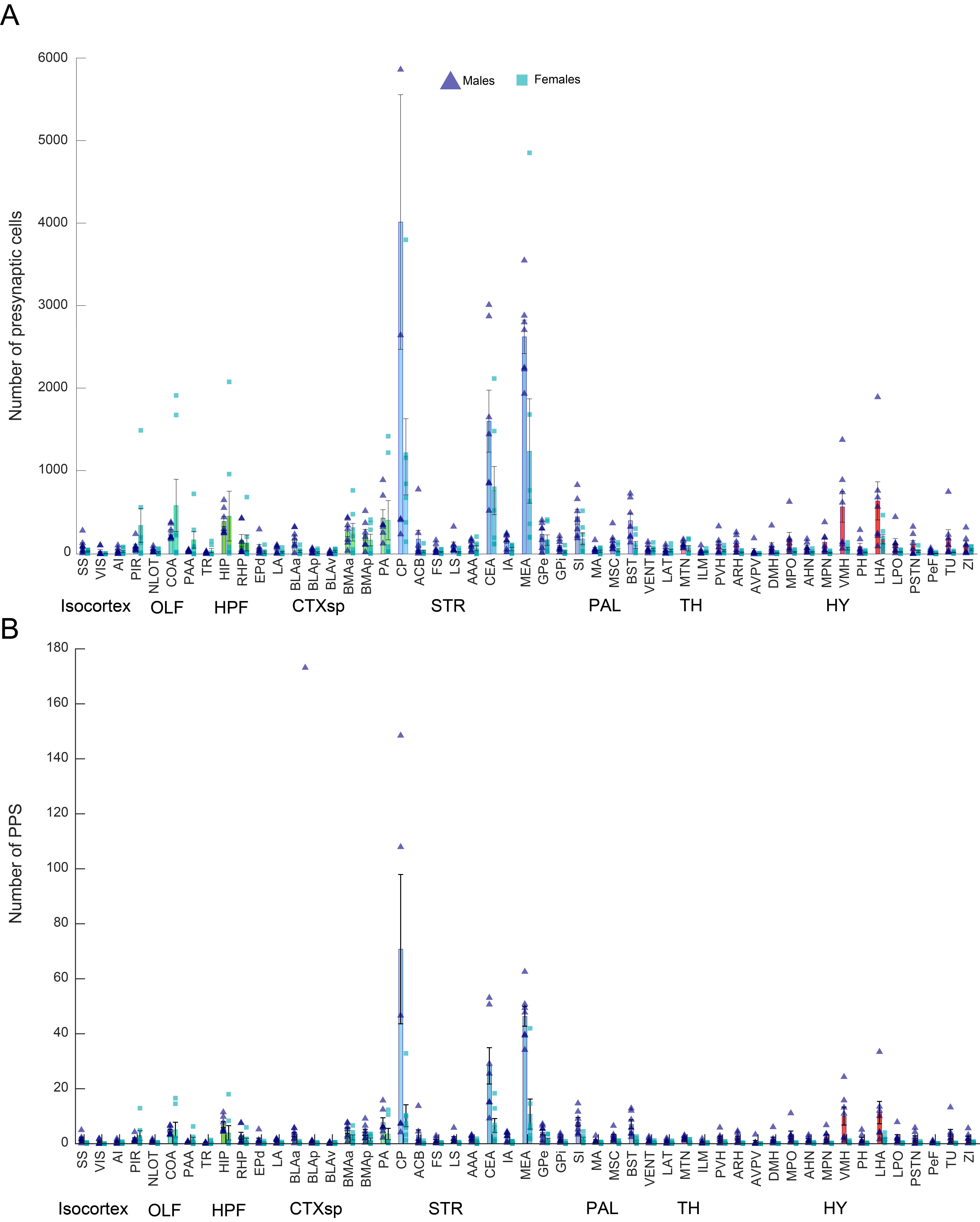

Supplement: Supplementary file 4 [file Image_1.JPEG]

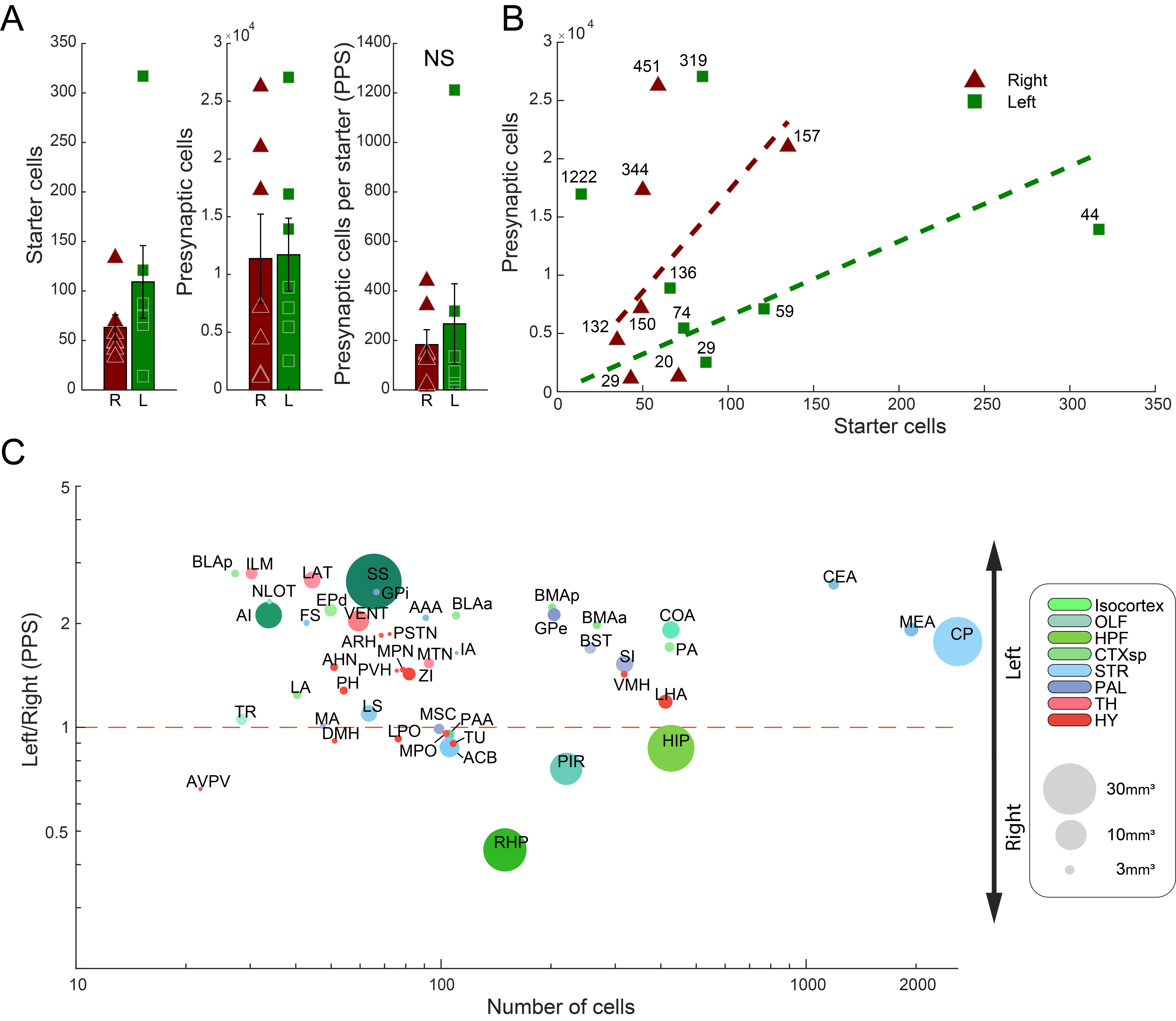

Supplement: Supplementary file 5 [file Image_2.JPEG]

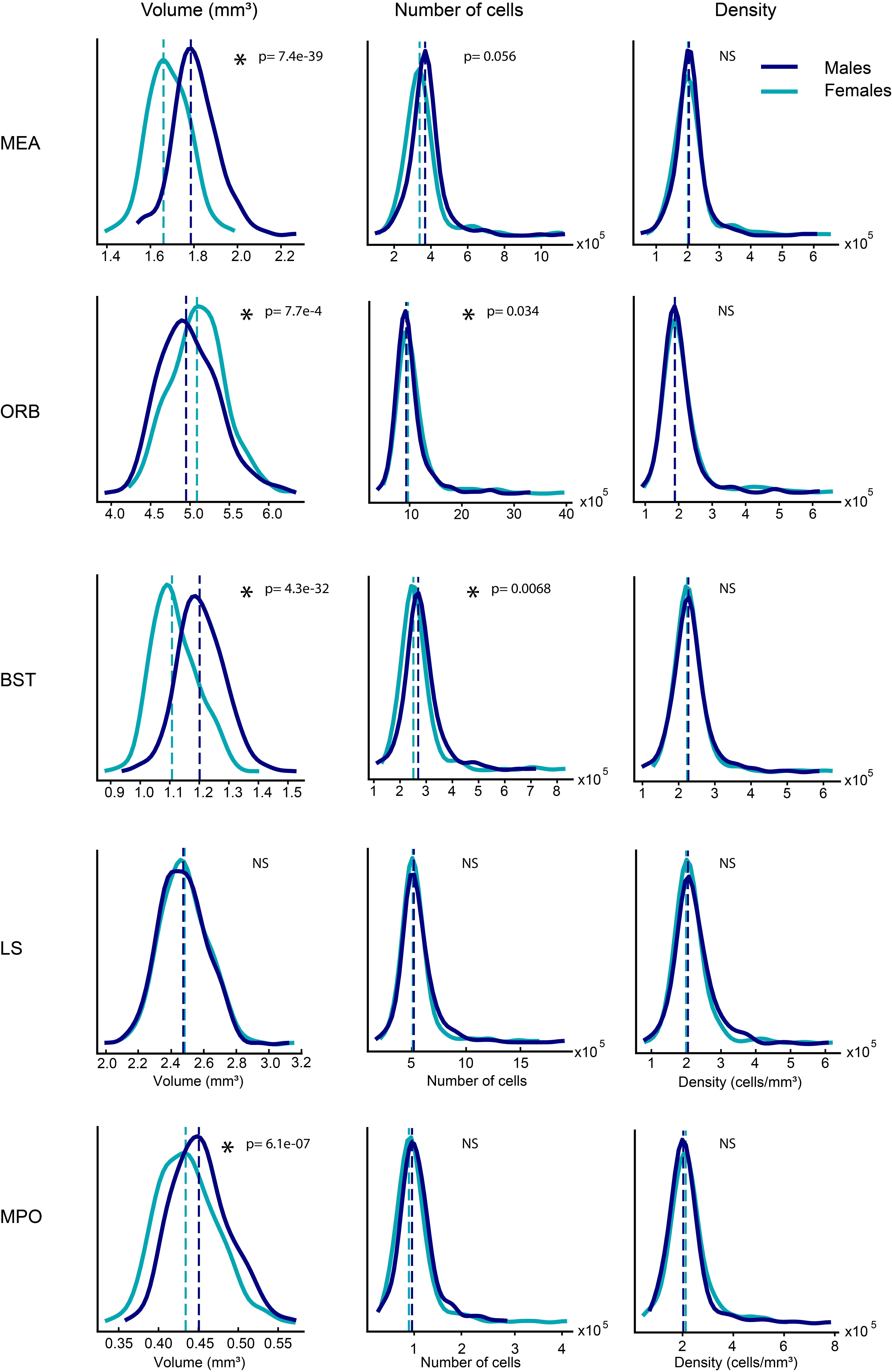

Supplement: Supplementary file 6 [file Image_3.JPEG]
